# Redox modulation of cellular stress response and lipoxin A4 expression by *Hericium Erinaceus* in rat brain: relevance to Alzheimer’s disease pathogenesis

**DOI:** 10.1186/s12979-016-0078-8

**Published:** 2016-07-09

**Authors:** A. Trovato, R. Siracusa, R. Di Paola, M. Scuto, M. L. Ontario, Ornella Bua, Paola Di Mauro, M. A. Toscano, C. C. T. Petralia, L. Maiolino, A. Serra, S. Cuzzocrea, Vittorio Calabrese

**Affiliations:** Department of Biomedical and Biotechnological Sciences, School of Medicine, University of Catania, Viale Andrea Doria 6, 95125 Catania, Italy; Department of Chemical, Biological, Pharmaceutical and Environmental Sciences, University of Messina, Messina, Italy

**Keywords:** Lipoxin A4, Heat shock protein70, Heme Oxygenase-1, Nutritional mushrooms, Alzheimer’s disease

## Abstract

**Background:**

There has been a recent upsurge of interest in complementary medicine, especially dietary supplements and foods functional in delaying the onset of age-associated neurodegenerative diseases. Mushrooms have long been used in traditional medicine for thousands of years, being now increasingly recognized as antitumor, antioxidant, antiviral, antibacterial and hepatoprotective agent also capable to stimulate host immune responses.

**Results:**

Here we provide evidence of neuroprotective action of *Hericium Herinaceus* when administered orally to rat. Expression of Lipoxin A4 (LXA4) was measured in different brain regions after oral administration of a biomass *Hericium* preparation, given for 3 month. LXA4 up-regulation was associated with an increased content of redox sensitive proteins involved in cellular stress response, such as Hsp72, Heme oxygenase −1 and Thioredoxin. In the brain of rats receiving *Hericium*, maximum induction of LXA4 was observed in cortex, and hippocampus followed by substantia Nigra, striatum and cerebellum. Increasing evidence supports the notion that oxidative stress-driven neuroinflammation is a fundamental cause in neurodegenerative diseases. As prominent intracellular redox system involved in neuroprotection, the vitagene system is emerging as a neurohormetic potential target for novel cytoprotective interventions. Vitagenes encode for cytoprotective heat shock proteins 70, heme oxygenase-1, thioredoxin and Lipoxin A4. Emerging interest is now focussing on molecules capable of activating the vitagene system as novel therapeutic target to minimize deleterious consequences associated with free radical-induced cell damage, such as in neurodegeneration. LXA4 is an emerging endogenous eicosanoid able to promote resolution of inflammation, acting as an endogenous “braking signal” in the inflammatory process. In addition, Hsp system is emerging as key pathway for modulation to prevent neuronal dysfunction, caused by protein misfolding.

**Conclusions:**

Conceivably, activation of LXA4 signaling and modulation of stress responsive vitagene proteins could serve as a potential therapeutic target for AD-related inflammation and neurodegenerative damage.

## Background

With the increased lifespan of the world’s population, it is estimated that about 80 million people will suffer from dementia by 2040 whereby AD accounted for almost 60 % of dementia cases [1–4]. Ageing, inexorable with decline in immune competence and onset of chronic inflammation, is one major causative factor associated to neurodegenerative diseases including dementia, Alzheimer’s disease (AD) and Parkinson’s disease (PD); atherosclerosis and stroke; diabetes; sarcopenia; and cancer [1–4]. Mushrooms have been used in traditional medicine for thousands of years [5, 6]. Many controlled studies have since investigated the long list of medicinal actions thought to be associated with extracts of these and other mushrooms, including antitumor, immunomodulatory, antioxidant, antiviral, antibacterial, and hepatoprotective effects [7]. Mushrooms are a rich source of polysaccharides and low molecular weight peptides [8–11], and many have been shown to stimulate host immune responses [12, 13]. The administration of complex mixtures of molecules of unknown concentrations is difficult to reconcile with current pharmaceutical practices involving highly purified compounds. The active ingredients may be unknown, making mushroom extracts very difficult to patent. Moreover, mushroom‑derived polysaccharides are complex molecules that cannot be synthesized, as the mass production of these compounds would require timely and costly extraction processes. As a result, many research efforts have focused on low molecular weight compounds, such as cordycepin [14]. Of the mushroom-derived therapeutics, polysaccharopeptides obtained from *Agaricus campestris*, *Pleurotus ostreatus* and *Coriolus versicolor* [15, 16], as well as *Hericium erinaceus* [17] are commercially the best established. While *Coriolus versicolor* is widely used to degrade recalcitrant organic pollutants such as pentachlorophenol (PCP) [18], *H. erinaceus*, has been used in traditional medical practices in China and Japan as to treat various human diseases. *H. erinaceus* mushroom is harvested, dried, ground, and made into medicinal preparations. The compounds isolated from fruiting bodies contain numerous biological activities, such as anti-tumor, hypolipidemic, hemagglutinating, cytotoxic, anti-microbial, endoplamic reticulum (ER) stress-suppressive, and antioxidant activities. In particular, it has been reported that hericenones and erinacines stimulate nerve growth factor (NGF) synthesis in cultured astrocytes. Erinacines have been isolated from cultured mycelia of *H. erinaceus*, and identified as one of diterpenoids. Hericenones have been isolated from the fruiting bodies of *H. erinaceus* and its molecular formula determined. In addition, studies from whole brain and cell cultures have shown that NGF affects the viability of cholinergic neurons, stimulating in the CNS the activity of enzymes, such as choline-acetyltransferase and acetylcholinesterase. These results are consistent with clinical finding indicating that *H. erinaceus* supplementation improves mild cognitive impairment, a condition where cholinergic neurons are centrally involved. However, despite the clinical relevance of *H. erinaceus* nutritional approaches*,* there have been only few studies elucidating mechanisms impacting defined brain physiopathological processes underlying the determinism of neurodegeneration involved in AD, as well as other types of dementia.

Recently, the involvement of neuroinflammation and microglial activation in the pathogenesis of AD has been emphasized by compelling evidence from basic and clinical research studies indicating that inflammation induced by amyloid beta (Aβ) is intimately associated with the development of AD neuropathology [19], in a scenario where Aβ activates microglia [20], the resident macrophages of the brain, and activated microglia may then promote neuronal injury through the release of proinflammatory and cytotoxic factors, sustaining further glial activation via a detrimental cycle where neuroinflammation and oxidative stress act in synergy, exacerbating the course of the disease [21]. Consistent to this notion, recent advances in knowledge of the mechanisms of inflammatory resolution have identified lipoxins as attractive therapeutic tools to treat diseases in which inflammation is involved [22]. Lipoxin (LXA4) is generated via the lipoxygenase pathway during cell-cell interactions in inflammatory conditions, whereas aspirin-triggered LXA4 (ATL), a molecule that displays the same anti-inflammatory activities as the native lipoxins, is generated after the acetylation of cyclooxygenase-2 and is more resistant to metabolic inactivation [23]. However, the ability of LXA4 signaling to modulate neuroinflammation and AD pathology in vivo has not been yet completely elucidated. Recent data from our laboratory [24] have demonstrated that administration for one month of a biomass preparation from *Coriolus versicolor* was able to modulate redox-dependently cellular stress response in rat. In view of the neuroinflammatory nature of Alzheimer’s disease pathogenesis and taken into account the therapeutic potential of mushroom nutritional approaches, the present study was conducted in order to gain insight into the possible neuroprotective role of *H. erinaceus* biomass preparation against the inflammatory process and to evaluate the impact of this intervention on cellular stress response mechanism operating in rat brain.

## Methods

### Chemicals

All reagents were from Merck (Germany) and of the highest grade available. *Hericium erinaceus* powder containing both mycelium and primordia (young fruit body) was supplied by Mycology Research Laboratories Ltd. (United Kingdom).

### *H. erinaceus* biomass preparation

*H. erinaceus* is found almost worldwide; however, its bioactivity varies depending on the habitat in which it grows. To eliminate these variations in the present study an established *H. erinaceus* strain was used which demonstrates rapid and aggressive colonization. Our *H. erinaceus* powder containing both mycelium and primordia (young fruit body) was supplied by Mycology Research Laboratories Ltd. This mushroom, cultivated into a biomass, is grown on a sterilized (autoclaved) substrate. The production process involves the inoculation of sterile organic edible grain with spawn from the mother culture. The fungus is thus allowed to colonize completely in an environment where the growth medium is aseptically kept. At the correct stage of development, corresponding to the maximum bioavailability the living biomass is aseptically air-dried, granulated, tested microbiologically and reduced in powder. In comparison to *H. erinaceus* extracts, biomass has the advantage of preserving all nutraceutical potential which is usually reduced with extracts or concentrates, including lyophilisation, and thus the activity of the product corresponds with the source mushroom, while being further intensified by utilizing the entire mycelium. The *H. erinaceus* biomass powder containing mycelium and primordia of the respective mushroom was used for experiments.

### Animals

The study was carried out using male Sprague-Dawley rats (200 to 230 g; Harlan, Nossan, Italy). Food and water were available *ad libitum*. The study was approved by the University of Messina Review Board for the care of animals. Animal care was in compliance with Italian regulations on the protection of animals used for experimental and other scientific purposes (DM116192) as well as with the relevant European Economic Community (EEC) regulations (OJ of EC L 358/1 12/18/1986).

### Experimental groups and treatments

Animals were randomly allocated into the following groups:

- ***Group 1***: Sham + Veh = vehicle solution (saline) was daily administered o.s. for 3 month (*N* = 10);
- ***Group 2***: Sham + *H. erinaceus* = same as the Sham + Veh group, but *H. erinaceus* powder (200 mg/kg body weight, soluble in saline o.s.) was administered daily by stomach gavage for the full experiment duration of 3 month (*N* = 10).

At the proper time points, animals were killed, brains quickly removed and dissected into the cerebral cortex, hippocampus, striatum and cerebellum, according to a standardized procedure, in a cold anatomical chamber and following a protocol that allows a maximum of 50 s time-variability for each sample across animals. SN was dissected from the deepest part of the interpeduncolar fossa. Samples from different rat brain areas, total brain, and from liver and kidney were homogenized for 2 min in 0.05 mol/L Tris–HCl buffer, pH 7.4 (1:9).

#### Sampling and lymphocyte purification

Blood was collected through cardiac puncture and added to tubes containing EDTA. Aliquots (2 ml) were utilized for lymphocytes purification, which was accomplished by using the Ficoll Paque System following the procedure provided by the manufacturer (GEHealthcare, Piscataway, NJ, USA).

### Western blot analysis

Inducible heat shock protein 70 (Hsp-70), heme oxygenase-1 (HO-1) and thioredoxin (Trx) protein levels were estimated by Western blot analysis which was accomplished as previously described [25]. Plasma samples were processed as such, while the isolated lymphocyte pellet as well as dissected brain regions brain were homogenized and centrifuged at 10,000 × *g* for 10 min. The supernatant was then used for analysis after determination of protein content. Proteins extracted for each sample, at equal concentration (50 μg) were boiled for 3 min in sample buffer (containing 40 mM Tris–HCl pH7.4, 2.5 % SDS, 5 % 2-mercaptoethanol, 5 % glycerol, 0.025 mg/ml of bromophenol blue) and then separated on a polyacrylamide mini gels precasting 4–20 % (codNB10420 NuSept Ltd Australia). Separated proteins were transferred onto nitrocellulose membrane (BIO-RAD, Hercules, CA,USA) in transfer buffer containing (0.05 % SDS, 25 mM Tris, 192 mM glycine and 20 % v/v methanol). The transfer of the proteins on the nitrocellulose membrane was confirmed by staining with Ponceau Red which was then removed by three washes in PBS (phosphate buffered saline) for 5 min each. Membranes were then incubated for 1 h at room temperature in 20 mM Tris pH 7.4, 150 mM NaCl and Tween 20 (TBS-T) containing 2 % milk powder and incubated with appropriate primary anti-HSP-72, anti- HO-1, and anti-Trx polyclonal antibodies (Santa Cruz Biotech. Inc.), overnight at 4 °C in TBS-T. The same membrane was incubated with a goat polyclonal antibody anti-beta-actin (SC 1615 Santa Cruz Biotech.Inc., Santa Cruz, CA, USA, dilution 1:1000) to verify that the concentration of protein loaded in the gel was the same in each sample. Excess unbound antibodies were removed by three washes are with TBS-T for 5 min. After incubation with primary antibody, the membranes were washed three times for 5 min. in TBS-T and then incubated for 1 h at room temperature with the secondary polyclonal antibody conjugated with horseradish peroxidase (dilution1:500). The membranes were then washed three times with TBS-T for 5 min. Finally, the membranes were incubated for 3 min with Super Signal chemiluminiscence detection system kit (Cod34080 Pierce Chemical Co, Rockford, IL, USA) to display the specific protein bands for each antibody. The immunoreactive bands were quantified by capturing the luminescence signal emitted from the membranes with the Gel Logic 2200 PRO (Bioscience) and analyzed with Molecular Imaging software for the complete analysis of regions of interest for measuring expression ratios. The molecular weight of proteins analyzed was determined using a standard curve prepared with protein molecular weight.

### Quantification of LXA4

LXA4 was analyzed using ELISA kit (CEB452Ge Cloud-Clone Corp). The assay was performed following the instructions of the manufacturer and measured by a microplate reader, by reading at 450 nm. For this assay, aliquots of plasma or tissues homogenates were used. Tissue samples after rinsed in to remove excess blood thoroughly and weighed before homogenization, were homogenized in ice-cold PBS (0.01 M, pH 7.2). The resulting suspension was sonicated and then centrifuged for 5 min at 5000 × g at 4 °C, and supernatants collected. Aliquots of standard, blank, plasma or tissue homogenates containing equal amounts of proteins were brought to a final volume of 50 μL and added into the appropriate wells, followed by addition of 50 μL of detection reagent A to each well. The plate was shaken gently before incubation for 1 h at 37 °C. The solution was aspirated and the wells were washed 3 times. The remaining liquid was removed completely by snapping the plate onto absorbent paper. 100 μL of detection reagent B were then added to each well followed by incubation for 30 min at 37 °C. After repeated washing (5 times), 90 μL of substrate solution was added to each well followed by incubation for 25 min at 37 °C. Finally, 50 μL of stop solution was added to each well, before measurement through reading at 450 nm.

### Statistical analysis

All results obtained were expressed in mean ± SEM. The data was analyzed by ANOVA to compare the different groups and considered significant with a *p* < 0.05. Each experiment was carried out in triplicate and repeated twice.

## Results

### Regional distribution of LXA4 expression in rat brain after treatment with H. erinaceus

**Fig. 1:**
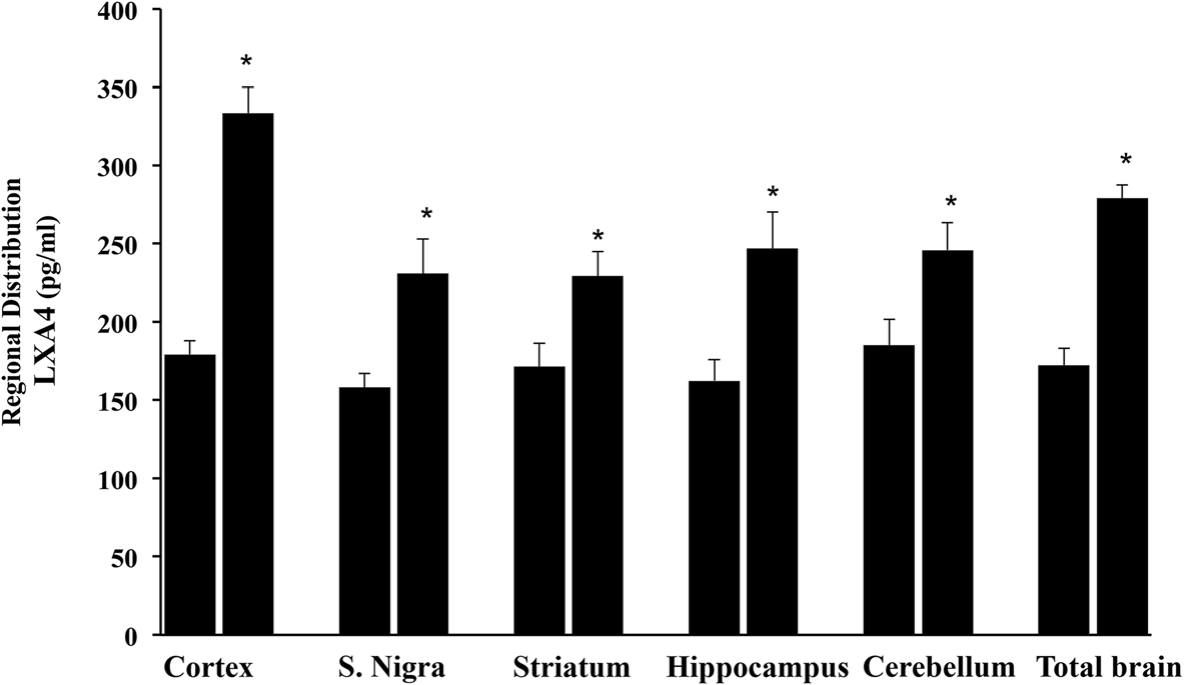
Regional distribution of Lipoxin A4 in rat brain. LXA4 protein levels in different brain regions and in total brain of control or *H. erinaceus*-fed rats. Values are expressed as mean ± SEM of three independent analyses on 10 animals per group. CX: cortex; Hp: Hippocampus; Cb: cerebellum; TB: Total Brain. *H. erinaceus*, given orally at the dose of 200 mg/Kg for 3 month. *P < 0.05 vs control

Expression of LXA4, in control animals and after administration of *H. erinaceus* biomass preparation, was investigated in different brain regions of rats, as well as in plasma and peripheral tissues, such as liver and kidney. Administration of *H. erinaceus* for 3 month at the oral daily dose of 200 mg/Kg induced an increase in the protein level of LXA4 in all brain regions examined. As shown in Fig. 1, highest levels of Hericium-dependent increase of LXA4 protein expression were observed in cortex, hippocampus, as well as in total brain, followed with a statistically significant difference by substantia nigra, striatum, and cerebellum.

### Mushroom-induced increase of LXA4 in peripheral tissues

**Fig. 2:**
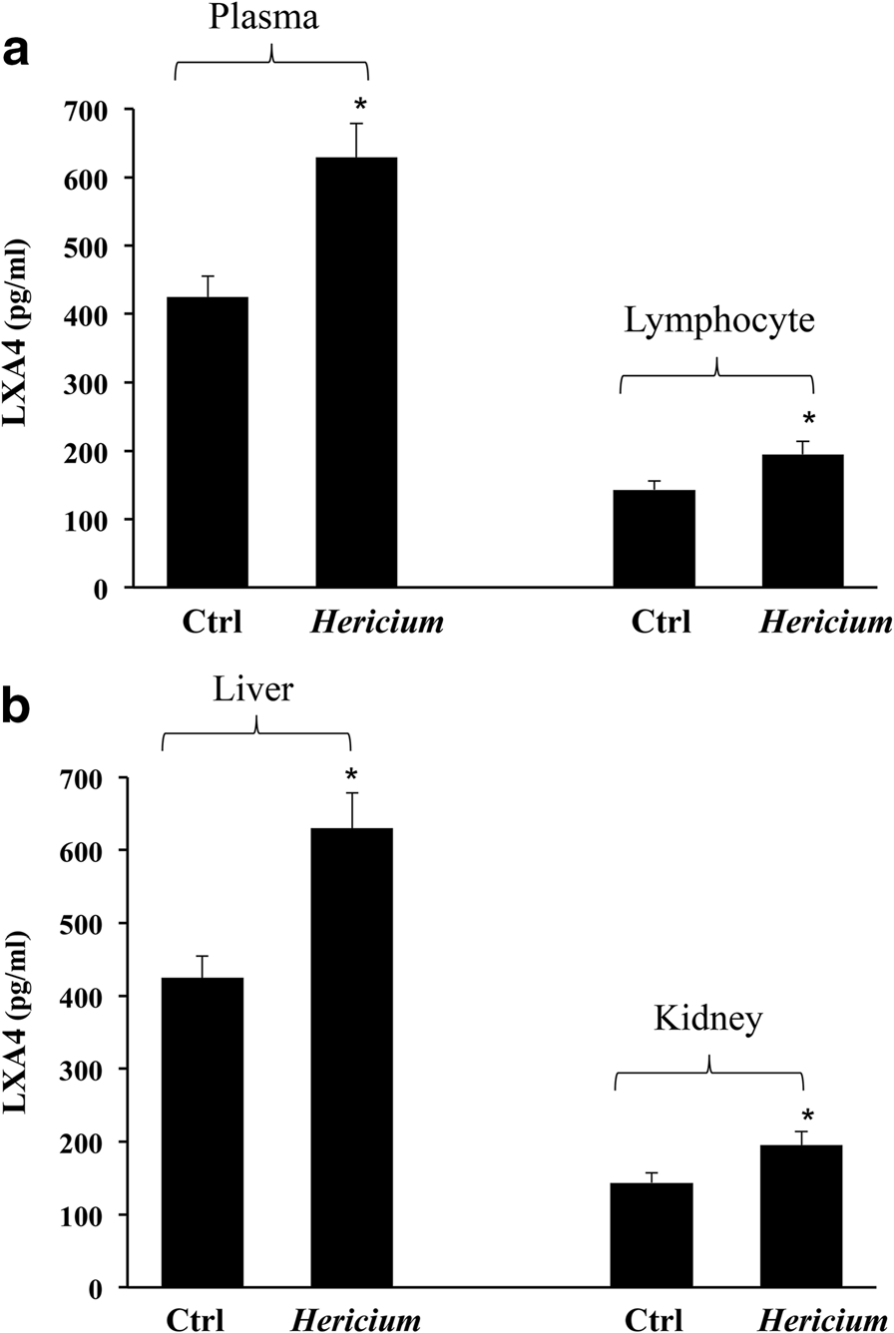
**a** Lipoxin A4 in rat blood. LXA4 levels in plasma and in lymphocytes from rats fed *H. erinaceus* biomass preparation as compared to control group. Data are expressed as mean ± SEM of 10 animals per group. *P < 0.05 vs controls. **b** Lipoxin A4 in Liver and Kidney. LXA4 levels in liver and kidney from rats fed *H. erinaceus* biomass preparation as compared to control group. Data are expressed as mean ± SEM of 10 animals per group. *P < 0.05 vs controls

In the group of animals receiving chronic administration of *H. erinaceus*, compared to untreated controls, brain changes in LXA4 protein were associated with a significant (*p* < 0.05) increase in the plasma and in lymphocytes (Fig. 2a), as well as in peripheral organs, such as liver and kidney, as illustrated in Fig. 2b.

### Modulation of HO-1, Hsp70 and Thioredoxin protein expression in rat brain after mushroom supplementation

**Fig. 3:**
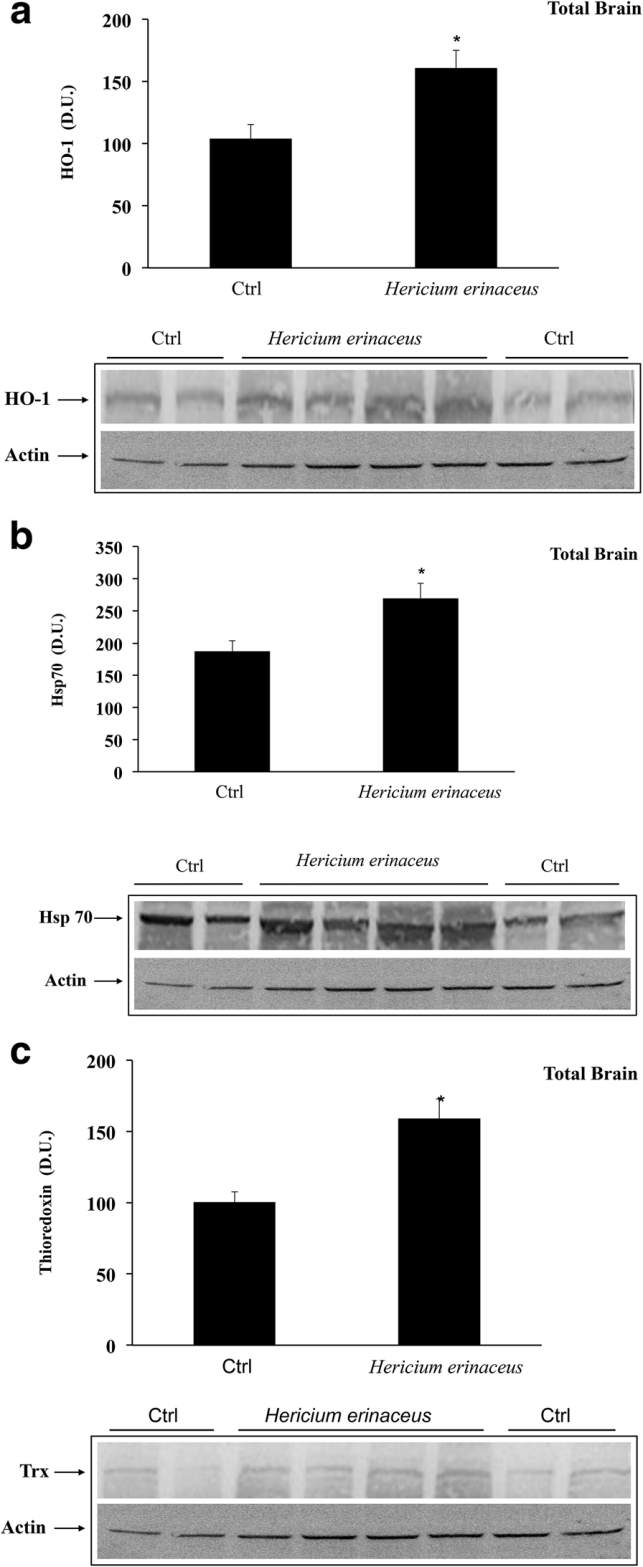
**a** Inducible heme oxygenase in Total Brain. HO-1 protein levels in the brain of rats fed *H. erinaceus* biomass preparation as compared to control group. Total brain homogenates from control and mushroom supplemented rats were assayed for HO-1 expression by Western blot. The bar graph shows densitometric values, expressed as mean standard error of mean of 3 independent analyses. *P* < 0.05 vs control. In the representative immunoblot shown, β-actin has been used as loading control. D.U., densitometric units. **b** Inducible Heat shock protein 70 in Total brain. Hsp70 protein levels in the brain of rats fed *H. erinaceus* biomass preparation as compared to control group. Total brain homogenates from control and mushroom supplemented rats were assayed for Hsp70 expression by Western blot. The bar graph shows densitometric values, expressed as mean standard error of mean of 3 independent analyses. *P* < 0.05 vs control. In the representative immunoblot shown, β-actin has been used as loading control. D.U., densitometric units. **c** Thioredoxin in Total brain. Trx protein levels in the brain of rats fed *H. erinaceus* biomass preparation as compared to control group. Total brain homogenates from control and mushroom supplemented rats were assayed for Trx expression by Western blot. The bar graph shows densitometric values, expressed as mean standard error of mean of 3 independent analyses. *P* < 0.05 vs control. In the representative immunoblot shown, β-actin has been used as loading control. D.U., densitometric units

**Fig. 4:**
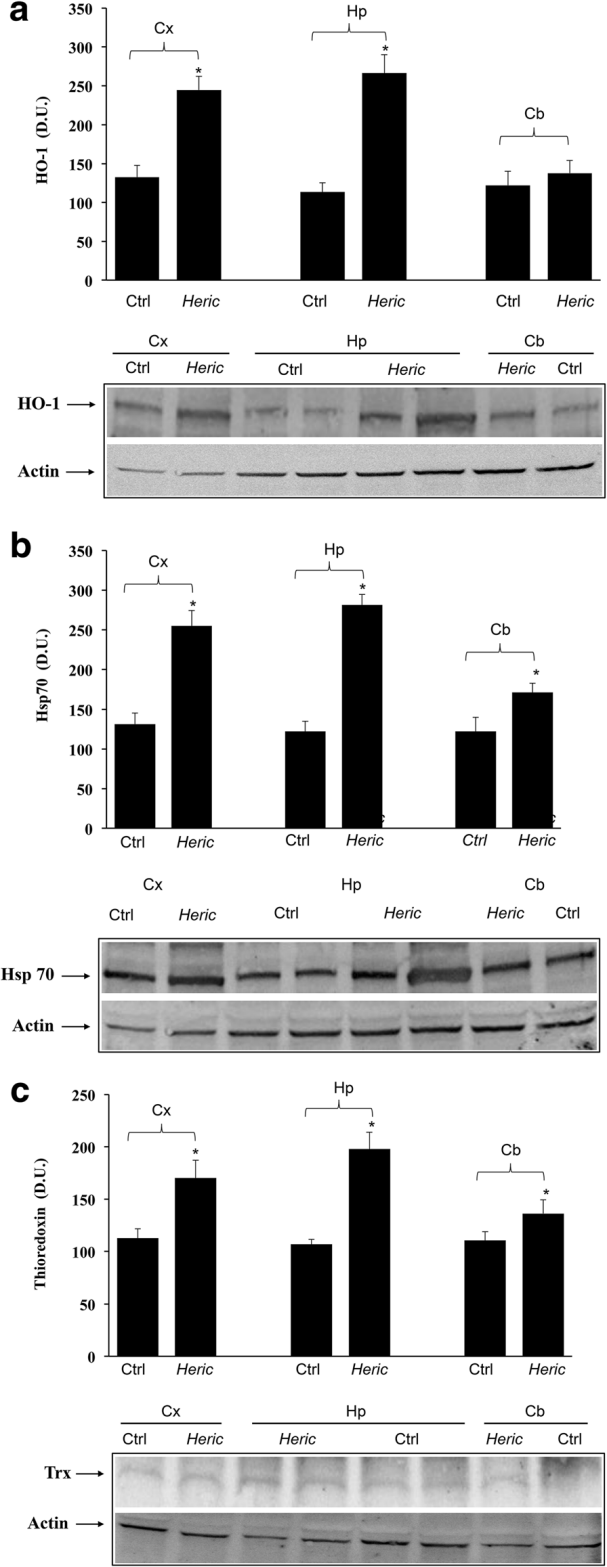
**a** Regional distribution of HO-1 in rat brain. HO-1 protein levels in different brain regions of rats fed *H. erinaceus* biomass preparation as compared to control group. Brain region homogenates from control and mushroom supplemented rats were assayed for HO-1 expression by Western blot. The bar graph shows densitometric values, expressed as mean standard error of mean of 3 independent analyses. *P* < 0.05 vs control. In the representative immunoblot shown, β-actin has been used as loading control. D.U., densitometric units. CX: cortex; Hp: Hippocampus; Cb: cerebellum. **b** Regional distribution of Hsp70 in rat brain. Inducible Hsp70 protein levels in different brain regions of rats fed *H. erinaceus* biomass preparation as compared to control group. Brain region homogenates from control and mushroom supplemented rats were assayed for Hsp70 expression by Western blot. The bar graph shows densitometric values, expressed as mean standard error of mean of 3 independent analyses. *P* < 0.05 vs control. In the representative immunoblot shown, β-actin has been used as loading control. D.U., densitometric units. CX: cortex; Hp: Hippocampus; Cb: cerebellum. **c** Regional distribution of thioredoxin in rat brain. Trx protein levels in different brain regions of rats fed *H. erinaceus* biomass preparation as compared to control group. Brain region homogenates from control and mushroom supplemented rats were assayed for Trx expression by Western blot. The bar graph shows densitometric values, expressed as mean standard error of mean of 3 independent analyses. *P* < 0.05 vs control. In the representative immunoblot shown, β-actin has been used as loading control. D.U., densitometric units. CX: cortex; Hp: Hippocampus; Cb: cerebellum

As reported in Fig. 3a and b, mushroom supplementation with *H. erinaceus* biomass preparation, resulted in up-regulation of brain cellular stress response proteins HO-1, and the inducible isoform of Hsp70. A representative Western blot, obtained probing total brain tissue homogenate with an antibody specific for the inducible isoform of heme oxygenase protein, is also shown (Fig. 3a). Western blot analysis of the inducible heat shock proteins 70 (Hsp72) revealed a significant increase in the brain of animals receiving *H. erinaceus* compared to control group. In the same figure a representative blot is reported (Fig. 3b). Figure 3c reports results from analysis of Trx protein expression in total brain of animals receiving *H. erinaceus*, as compared to controls. As shown, Trx protein increases significantly after *Hericium* supplementation. These results were also confirmed when measuring HO-1, Hsp70 and Trx protein expression in different brain regions of animals supplemented with *H. erinaceus*, as compared to the control group (Fig. 4a-c). Figure 4a shows a significant increase of HO-1 protein levels induced by this nutritional mushroom in the cortex and in the hippocampus, whereas this increase was not statistically significant *vs* controls in the cerebellum. In the same figure a representative Western blot, obtained probing the different brain regions for HO-1 is reported (Fig. 4a).

Figure 4b shows increased Hsp70 protein expression after *H. erinaceus* administration, which was significant, compared to control animals, in the cortex, hippocampus and cerebellum. In the same figure a representative Western blot, obtained probing the different brain regions for inducible isoform of Hsp70 is reported (Fig. 4b). Consistent with HO-1 and Hsp70 data, analysis of thioredoxin protein revealed that *H. erinaceus* biomass supplementation upregulated this protein levels in the brain regions of cortex, hippocampus and cerebellum (Fig. 4c), which was statistically significant in all brain regions examined (*P* < 0.05 vs control).

**Fig. 5:**
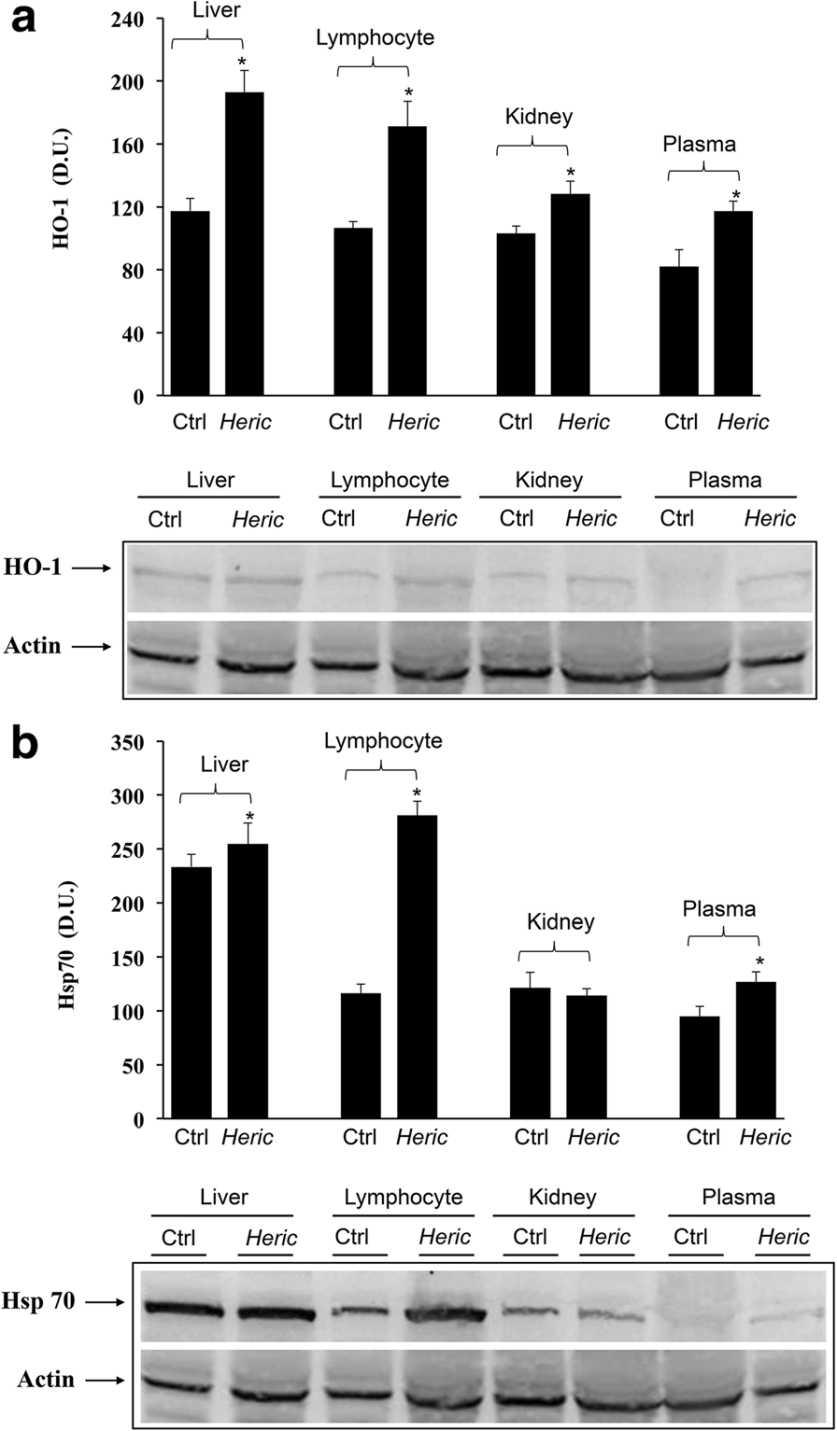
**a** Inducible heme oxygenase-1 in peripheral tissues. HO-1 protein levels in liver, lymphocyte, kidney and plasma from rats fed *H. erinaceus* biomass preparation as compared to control group. Samples from control and mushroom supplemented rats were assayed for HO-1 expression by Western blot. Bar graph shows the values expressed as mean standard error of mean of 3 independent analyses. *P* < 0.05 vs control. In the representative immunoblot shown, β-actin has been used as loading control. D.U., densitometric units. **b**. Inducible heat shock protein 70 in peripheral tissues. Inducible Hsp 70 protein levels in liver, lymphocyte, kidney and plasma from rats fed *H. erinaceus* biomass preparation as compared to control group. Samples from control and mushroom supplemented rats were assayed for Hsp70 expression by Western blot. Bar graph shows the values expressed as mean standard error of mean of 3 independent analyses. *P* < 0.05 vs control. In the representative immunoblot shown, β-actin has been used as loading control. D.U., densitometric units

**Fig. 6:**
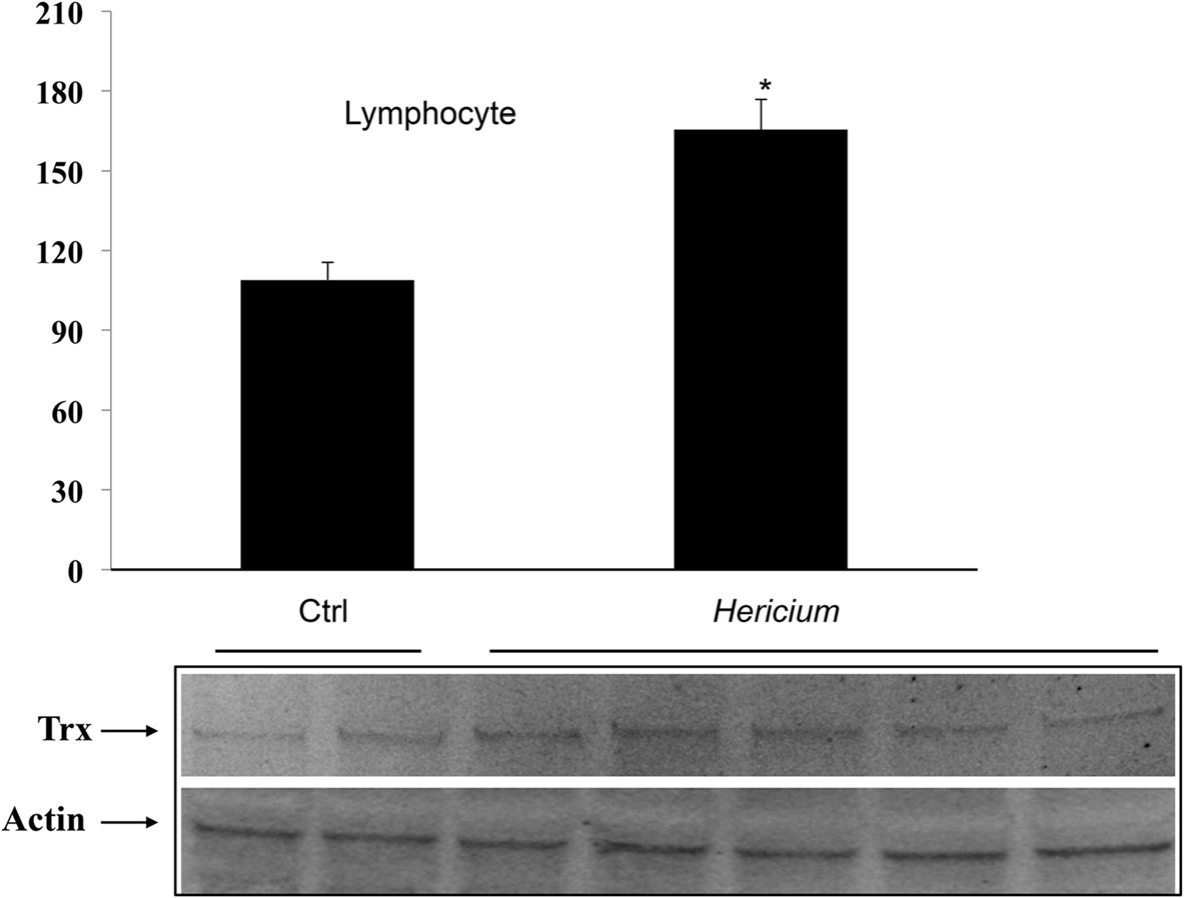
Inducible Thioredoxin in peripheral tissues. Trx protein levels in lymphocyte from rats fed *H. erinaceus* biomass preparation as compared to control group. Samples from control and mushroom supplemented rats were assayed for Trx expression by Western blot. Bar graph shows values expressed as mean standard error of mean of 3 independent analyses. *P* < 0.05 vs control. The representative immunoblot shows Trx and β-actin, which has been used as loading control. D.U., densitometric units

We then investigated whether administration of *H. erinaceus* would have affected systemic as well as peripheral stress response. As illustrated in Fig. 5a a significant induction of HO-1 protein was observed in the plasma, lymphocytes and, respectively, liver and kidney of *H. erinaceus* fed rats, as compared to untreated rat group. Representative blots are reported in the same figure. Figure 5b shows a parallel increase in Hsp70 protein expression, which was found in plasma, lymphocytes and liver, but not in the kidney of *H. erinaceus* fed animals. Consistent to the notion that mushroom biomass supplementation modulates stress responsive vitagenes we also found an increased protein expression of the redox sensitive Trx in lymphocyte of *H. erinaceus* fed rat, which was significantly higher respect to basal expression of this redox protein measured in control, untreated animals (Fig. 6). A representative Western blot, obtained probing total brain tissue homogenate with an antibody specific for the Trx protein is shown in the same figure.

## Discussion

AD has gained widespread attention because of its high economic costs, which have reached $400 billion per year in the USA alone, as well as the social costs, which are more difficult to quantify [26]. AD-related pathological markers include a progressive death of neurons in specific areas with an accumulation of intracellular neurofibrillary tangles (NFTs) and extracellular depositions of amyloid plaques (APs). NFTs are composed of the misfolded hyperphosphorylated microtubule-associated protein Tau (MAPT or Tau), whereas APs are extracellular deposits of misfolded and aggregated amyloid-beta peptides (Aβ) [27, 28]. Because both NFTs and APs are persistently found in areas with severe neuronal death, these proteins were considered to be the main cause of neuronal loss and the emergence of dementia, which is a crucial symptom of AD; however, numerous drug trials based on these proteins have failed to provide a useful AD therapy [28]. A post-mortem study demonstrated that the misfolded protein accumulation is a shared pattern in many neurodegenerative diseases, including AD [25], concurring to the conclusion that accumulation of misfolded proteins is a prominent potential cause of neurodegeneration in AD [29]. Recently, the involvement of neuroinflammation and microglial activation in the pathogenesis of AD has been emphasized by compelling evidence from basic and clinical research studies indicating that inflammation induced by Aβ is intimately associated with the development of AD neuropathology [19]. Relevant to the central role of neuroinflammation in AD pathgenesis, are recent advances in knowledge of the mechanisms of inflammatory resolution, identifying lipoxins as attractive therapeutic tools to treat diseases in which inflammation is involved [22]. LXA4 is generated via the lipoxygenase pathway during cell-cell interactions in inflammatory conditions, whereas aspirin-triggered LXA4 (ATL), a molecule that displays the same anti-inflammatory activities as the native lipoxins, is generated after the acetylation of cyclooxygenase-2 and is more resistant to metabolic inactivation [23]. Lipoxins potentiate inflammatory resolution by means of potent agonistic actions at the G-protein coupled receptor, termed LXA4 receptor (ALX/FPR2). Activation of ALX by LXA4 reduces many endogenous processes, such as neutrophil and eosinophil recruitment and activation, leukocyte migration, NF-kB translocation, and chemokine and cytokine production [22]. Likewise, evidence shows that LXA4 signaling primes macrophages for chemotaxis and enhances phagocytosis of microorganisms and apoptotic cells. In the nervous system, LXA4 protects neurons against experimental stroke and Aβ_42_ toxicity by modulating inflammation. In addition, lipoxins inhibit inflammatory pain processing through their actions on astrocytic activation in the spinal cord [30, 31]. However, the ability of LXA4 signaling to modulate neuroinflammation and AD pathology in vivo has not been yet completely elucidated.

Mushrooms provide a great potential as a polypharmaceutic drug because of the complexity of their chemical contents and different varieties of bioactivities. If available evidence suggests anti-oxidants, anti-tumor, antivirus, anti-cancer, anti-inflammatory, immune modulating, anti-microbial, and anti-diabetic activities from mushrooms [32], however, contrarily to plant herbal medicines, which are widely explored and relatively more advanced, the brain and cognition health effects of mushrooms are in the early stages of research. Here, by extending previous finding on nutritional approaches to neuroinflammation, we provide experimental evidence that administration of *H. erinaceus* for 3 month to rats results in up regulation of vitagenes, in particular Hsp70, HO-1 and Trx, an effect associated with increased synthesis of LXA4 in different brain regions of rat. This latter, an endogenous eicosanoid, is emerging as an important resolvin, a class of compounds endowed with the capability to promote resolution of inflammation, therefore suggesting that nutritional modulation with *H. erinaceus*, through redox-dependent vitagene network might activate endogenous “braking signal” processes impacting the inflammatory process. We also provide evidence of neuroprotective action of *H. erinaceus* when administered orally to rat. Expression of LXA4, measured in different brain regions after oral administration of a biomass *H. erinaceus* preparation for 3 month increased significantly in all brain regions examined, as compared to control group of animals, particularly in cortex and cerebellum, followed by substantia Nigra, striatum and cerebellum. LXA4 up-regulation was associated with an increased content of redox sensitive proteins involved in cellular stress response, such as Hsp72, HO-1 and Trx. We show that SN exhibited lower LXA4 content respect to other brain regions examined, both in control and mushroom stimulated animals. This finding is relevant to AD and PD pathogenesis, particularly to theories connecting aging and neuronal degeneration with oxidative damage. SN neurons are depleted during physiological aging and even more so in all neurodegenerative processes associated with Parkinsonian symptoms. [33–37]. In addition, we demonstrate that *H. erinaceus* treatment resulted in a significant increase of LXA4 in most of the brain regions examined and modulated expression of cytoprotective proteins, such as HO-1, Hsp70 and Trx. Our results are consistent with recent evidence obtained in mice, showing neuroprotection by *H. erinaceus* on Ab25–35 peptide-induced cognitive dysfunction [38, 39]. In this study the powder of *H. erinaceus* was mixed with a normal powdered diet and the Ab25–35 peptide was administered by intracerebroventricular injection. The results revealed that H. erinaceus prevented impairments of spatial short-term and visual recognition memory induced by Ab25–35 in mice. Furthermore, human trials with *H. erinaceum* derivatives also have showed promising results in patients with dementia based on Revised Hasegawa Dementia Scale (HDS-R) [40].

Our results indicating that nutritional modulation of critical proteins involved in brain stress tolerance can be achieved via supplementation with a well characterized strain of *H. erinaceus* are relevant to those theories connecting proteome control quality failure with age-associated neurodegenerative diseases. Consistent to this notion, in AD pathology, the accumulation of APs composed of Aβ aggregates and neurofibrillary tangles NFTs composed of misfolded Tau proteins, accumulation of these proteins as consequence of faulty protein quality control mechanisms, is associated with a deficit in those mechanisms participating to induction of cytoprotective proteins (Hsps) or, more in general, involved in the cellular pathways of stress tolerance. It is conceivable that in these conditions administration of *H. erinaceus* mushroom, which increases the redox potential associated with induction of vitagenes, may help vulnerable neurons to resist to proteotoxic insults and hence to apoptotic neurodegeneration. This is furtherly corroborated by the finding indicating that restoration of normal proteostasis is crucial for neuronal survival [41].

The molecular chaperone Hsp70 protects cells from injury by binding damaged proteins under stressful situations. Members of the 70 kDa-heat shock protein family (Hsp70s) are, in their function as molecular chaperones, involved in folding of newly synthesized proteins and refolding of damaged or misfolded proteins, as well as in assembly and disassembly of protein complexes. All human Hsp70s have highly conserved domain structures [42]. They consist of an N-terminal ATPase domain, a middle region and an N-terminal peptide binding domain. However, they differ in expression patterns, cellular localization and function. There are Hsp70’s specifically located in the endoplasmatic reticulum (Grp78, also known as BiP) and in the mitochondria (Grp75, also known as mortalin). However, the members which are mainly located in the cytosol and nucleus are the heat shock cognate protein 70 (Hsc70) and the heat shock protein 70 (Hsp70). Cellular stress often leads to protein unfolding and, therefore, to increased protein hydrophobicity, which may result in the formation of toxic protein aggregates [42]. As recently demonstrated, Hsp70 expression is induced under the mild oxidative stress conditions, when oxidative damage to proteins leads to their unfolding [43], and the heat shock response is activated driving increases in the expression of molecular chaperones, which reaches about two-fold the baseline levels [43]. Although HSP’s can refold mildly disordered proteins, it is clear that HSP’s are not able to repair covalently-modified oxidized proteins or to reverse oxidative protein modifications, which results in increased protein hydrophobicity, as triggering signal for the activation of a highly regulated and rapid series of events, called the ‘heat shock response’ (HSR). Heat shock transcription factor 1 (HSF1) is bound to a complex of heat shock proteins (Hsps), such as Hsp70 and Hsp90, during non-stressed conditions and, therefore, kept in an inactive state. When Hsps recognize hydrophobic patches of damaged and unfolded proteins, the Hsps dissolve from the complex with HSF1 in response to cellular stress. This event is followed by HSF1 trimer formation, which further leads to the activation and translocation of the transcription factor into the nucleus, where the trimer binds to the heat shock gene promoters, the so called heat shock elements (HSEs). This leads to the fast expression of Hsps [44]. Moreover, the heat shock genes do not contain introns, which further accelerates their expression. An unfolded protein that binds to Hsp70 may be either refolded into its native non-toxic conformation and then released, or may stay bound by Hsp70 to protect non-damaged molecules. Since most oxidative protein modifications are not repairable due to their covalent nature, the majority of oxidized proteins are degraded by the proteasomal system.

## Conclusions

Our finding can open up new neuroprotective strategies, as interventions aiming at inducing the vitagene defense system mechanism, including Hsps, HO-1, thioredoxin and lipoxin A4, can represent a therapeutic target to minimize the deleterious consequences associated to oxidative stress, such as in brain aging and neurodegenerative disorders.

## Abbreviations

AD, Alzheimer’s disease; APs, amyloid plaques; ATL, aspirin-triggered LXA4; Aβ, amyloid-beta peptides; EEC, European Economic Community; ER, endoplamic reticulum; *H. erinaceus*, *Hericium. erinaceus*; HDS-R, Revised Hasegawa Dementia Scale; HO-1, heme oxygenase-1; Hsc70, heat shock cognate protein 70; HSEs, heat shock elements; HSF1, Heat shock transcription factor 1; Hsp-70, heat shock protein 70; HSR, heat shock response; LXA4, Lipoxin A4; MAPT or Tau, microtubule-associated protein Tau; NFTs, intracellular neurofibrillary tangles; NGF, nerve growth factor; PCP, pentachlorophenol; PD, Parkinson’s disease; St, striatum; Trx, thioredoxin
